# Implementation research and human-centred design: how theory driven human-centred design can sustain trust in complex health systems, support measurement and drive sustained community health volunteer engagement

**DOI:** 10.1093/heapol/czaa129

**Published:** 2020-11-06

**Authors:** Mary B Adam, Joy Minyenya-Njuguna, Wilson Karuri Kamiru, Simon Mbugua, Naomi Wambui Makobu, Angela J Donelson

**Affiliations:** 1 AIC Kijabe Hospital, Kijabe, PO Box 20, 00220, Kenya; 2 Donelson Consulting, 3173 Adelle Place, Tucson, Arizona, 85749, USA

**Keywords:** Human-centred design, implementation research, trust, measurement, health system, trust relationships, reciprocity, process evaluation

## Abstract

Human-centred design (HCD) can support complex health system interventions by navigating thorny implementation problems that often derail population health efforts. HCD is a pragmatic, ‘practice framework’, not an intervention protocol. It can build empathy by bringing patient voice, user perspective and innovation to construct and repair pieces of the intervention or health system. However, its emphasis on product development and process change with fixed end points has left it as an approach lacking explanatory power and reproducible measurement. Yet when informed by theory, the tremendous innovation potential of HCD can be harnessed to drive sustainability, mediate implementation problems, frame measurement constructs and ultimately improve population-level health outcomes. In attempting to mine, the potential of HCD we move beyond the pragmatic ‘how it works’, to the theoretical question, ‘why it works’. In doing so, we explore a more fundamental human question, ‘How can participation and engagement be sustained for impact in close to the community health systems?’ In this exploration, we illustrate the power of HCD by linking it to our theory of trust building. The research method we utilize is that of a longitudinal process evaluation. We leverage the heterogeneity of five community health units from the diverse setting (rural, peri-urban slum) to better understand what works for whom and in what context by tracking 21 groups of community health volunteers (CHVs) over 12 months. We report results with a focus on the outlier case failure to illustrate the contrast with common features of sustained CHV engagement, where recurrent reciprocal cycles of trust building are demonstrated in the successful implementation of action plans in plan-do-study-act cycles for improvement. All was accomplished by CHVs with no external funding. We conclude by discussing how HCD could be unleashed if linked to theoretical frameworks, increasing ability to address implementation challenges in complex health systems.

**KEY MESSAGES**

- Human-centred design (HCD) brings an innovation platform with an opportunity for all levels of the health system.
- When informed by theory, the tremendous innovation potential of HCD can be harnessed to drive sustainability, mediate implementation problems and frame measurement constructs.
- We illustrate the power of HCD by linking it to a theory of trust building in health systems and demonstrate how it can address context-specific implementation issues and drive sustainability.

## Introduction

Human-centred design (HCD) can assist community health partnerships in navigating thorny implementation problems that often derail community health efforts. HCD is a pragmatic, ‘practice framework’, not a protocol rooted in theory (Roberts *et al.*, 2016). It can build empathy by bringing patient voice, user perspective and innovation to construct and repair pieces of the health system. The health system encompasses interactions among diverse sets of stakeholders, such as where healthcare is delivered when a community member is referred to the facility, or where facility staff need permission from higher-level management to enact certain policies and procedures. These are all points within a top-down hierarchy, where those closer to the household level tend to be lowest in power and decision-making opportunity.

HCD provides the opportunity to strengthen human agency at the individual level and amplify the voice of those lower in power through its valuable framework, tools and processes. However, its emphasis on product development and process change with fixed end points has left it as an approach lacking explanatory power and reproducible measurement (Bazzano *et al.*, 2017). We argue that when informed by theory, HCD has potential to drive sustainability, to mediate implementation problems and to frame measurement constructs in complex health systems and in so doing improve population-level health outcomes.

In demonstrating the power of linking HCD to theory, we use a theory of trust building. We choose this theory because trust is required for successful implementation of complex interventions in health systems. Trust is indispensable for health systems to function well because they are largely relational (Langley, 1999; Gilson, 2003; Gwebu *et al.*, 2007; Topp and Chipukuma, 2016). Trust improves collaborative decision-making for effective and sustainable partnerships and citizen accountability (Hicks *et al.*, 2012; Rifkin, 2014; Dadwal *et al.*, 2017). Implementation challenges for interventions illustrate how behavioural change, like trust building, is a human process, rooted in a relationship within a specific cultural context. Understanding science is not enough to ensure uptake (Kumar *et al.*, 2010, 2015).

In linking HCD to a theory of trust building, we explore a more fundamental human question, ‘How can participation and engagement be sustained in close to the community health systems?’

First, we describe the utility and limitations of HCD. Next, we introduce a process-based theory of trust building and link it to an HCD framework to demonstrate how close to the community health systems can facilitate innovation for sustained action. Third, we show how our theory of trust building operates in practice, using a longitudinal process evaluation approach, leveraging the heterogeneity of multiple case studies. We conclude by discussing how HCD could be better harnessed if linked to theoretical frameworks, thus addressing its ability to implement interventions in complex health systems.

## Human-centred design

The influence of HCD approaches is growing in health systems research (Diechmann and van der Heijde, 2016; Andresen and Potter, 2017; Kim *et al.*, 2017). As a ‘practice framework’, HCD integrates a three-part cyclical process of helping stakeholders derive inspiration (understanding the experiences, needs and challenges of users of community resources), produce ideation (creating ideas and solutions) and conduct the implementation of community health strategies (Hendricks *et al.*, 2018). HCD seeks to elicit empathy for users so as to understand how different sets of people experience health and see and address challenges and solutions within their context. It does so through the use of diverse and collaborative teams, working on action-oriented rapid prototyping based on user derived insights rather than from top-down hypotheses (Roberts *et al.*, 2016). As such, design thinking seeks to enact quick, iterative ‘Plan-Do-Study-Act’ cycles to make change happen. This facilitates stakeholders learning more about the problem or solution as they go.

In the context of community health systems, HCD provides a platform to incorporate the voice and experience of the user when designing health services, systems or products (Bazzano *et al.*, 2017). It enables stakeholders in close to the community health systems to engage fully, rather than participate as passive agents. This is valuable in environments where top-down structures prevail and/or shame-based norms tend to predominate. With its emphasis on experimentation and on iteration, HCD provides rapid cycles for testing success or failure, allowing stakeholders to experiment without condemnation, offering more independent agency and potentially less dependence on patrons or donors.

HCD differs from some of the more common methodological approaches employed in health systems. Among the most popular of these is a quality improvement (QI). QI practitioners take a problem-focused approach to healthcare improvement, often redesigning processes to achieve new levels of performance (Taylor *et al.*, 2014; Horwood *et al.*, 2015; Horwood *et al.*, 2017). QI looks to evidence-based medical perspectives to solve specific health problems with pre-defined outcomes (such as implementing a set protocol to reduce blood stream infections). While both QI and HCD approaches favour redesigning processes and enacting Plan-Do-Study-Act cycles to obtain change, HCD takes a step beyond QI. HCD practitioners seek to push beyond pre-determined outcomes because they recognize co-producing solutions with end users requires different and often messy trajectories of engagement for sustainability and cultural relevance. HCD’s ability to address ambiguity or flexibility in the process allows a different path into addressing a health challenge, one that focuses on unpacking and testing potential solutions through multiple iterations. Therefore, QI tends to be a poor fit for processes that puts community members and local health leaders at the centre of changing action strategies.

While valuable for engaging participation, HCD frameworks are limited in the ability to generate sustained change. They have rarely been harnessed to address and mediate implementation problems or to frame measurement outcomes. This is because with standard uses of HCD, sustainability is limited by the nature of product development or process improvement cycles, which have short-term product-oriented perspective related to health outcomes. In his review of published HCD research in global health over the past decade, Bazzano *et al.* (2017) found very limited empirical evidence for HCD’s impact on mid-term (individual behaviour) change, such as mental health, self-reliance/esteem and health behaviours. There is even a weaker link between these outcomes and longer range health and well being. Moreover, the linkage was missing between these individual behaviours and community-level, public health outcomes. HCD falls short of linking short-term to longer-term measurement constructs because its framework does not seek to offer causal explanations for change, or to link these within an individual-to-community context (Bazzano *et al.*, 2017; Moore *et al.*, 2019). Yet, understanding causal assumptions and processes are particularly important in the implementation of a health intervention, especially in health community engagement models where collaboration and shared leadership are important (Eder, 2013; Moore, 2019).

HCD’s perspective is limited in that it was developed from a very pragmatic, practice-framework approach. It has been commonly used as a deep dive for information in order to support decision-making, especially in healthcare. It does, however, have the potential to address social mechanisms, where stakeholders’ activities, events and choices interact within the context of a process model. We present such a model later in this paper as an example where HCD leveraged power when linked to a reciprocity-based theory of trust building. That is, social mechanisms linked to trust theory, operating within a pragmatic HCD framework, can support sustained engagement.

## A process-based theory of trust building

Exploring sustained engagement for long-term health outcomes requires theory-building and testing. As we have described elsewhere (Adam and Donelson, 2018b), a process-based theory of reciprocal relationships for trust building is helpful for understanding reasons for sustained engagement in built within close to community health systems.

**Figure 1:**
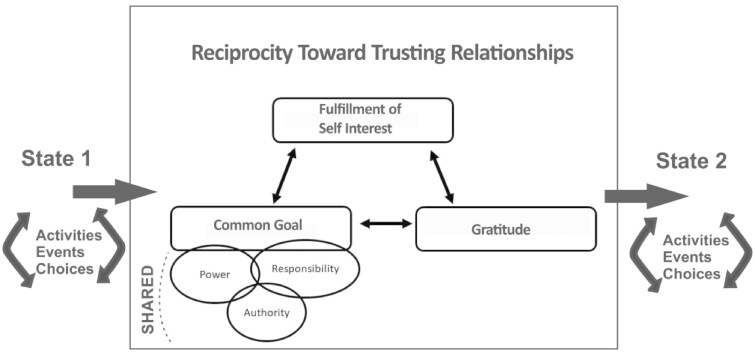
Reciprocity towards trusting relationships.

Figure 1 shows the building blocks for process-based trust building. We follow Langley’s (Langley, 1999; Langley *et al.*, 2013) general process model of the interaction of events, activities and choices. Every process model has interrelated events and activities that shape choices, in moving from one state to another. As shown in Figure 1, these are influenced by the mediating effect of reciprocal relationships, which form the building blocks of trusting relationships. Reciprocal relationships are influenced by three interrelated elements: the achievement of common goals, the fulfilment of shared self-interests and expression of gratitude/indebtedness. The interaction among these three, in a reciprocity cycle that mirrors a PDSA cycle, enhances agency in participants and changes the initial process state.

In Figure 1, common goal refers to the pursuit of three principles: shared power, shared responsibility and shared authority. This provides stakeholders motivation for engagement, focusing co-production of knowledge and action at the intersection of the end-user/health system interface rather than solely within the traditional health systems hierarchy (Langley *et al.*, 2018). Organizational change theorists (Kotter and Schlesinger, 1979, Langley *et al.*, 2018), who have influenced the field of organizational management with their principles, claim these are important because power enables less influential actors within a system to leverage their influence with those higher up the power ladder, responsibility enables less influential actors to solve their own problems through their own projects, and authority gives less influential actors the ability to act.

Self-interest keeps stakeholders at the table. Organizational psychologists argue that individuals are motivated by self-interests that are intrinsic (such as self-worth and belonging) and extrinsic (such as reputation and tangible rewards) (Wasko and Faraj, 2005). Both are important to articulate and then meet for continued reciprocal transactions. This articulation can build empathy, or what HCD practitioners define as an understanding of how others experience things from their point of view (Gasparini, 2015). Within a health systems context, these include motivational positions and aspirations of end users and stakeholders. Table 1 illustrates how intrinsic and extrinsic self-interests might be articulated within a close to the community health system.

**Table 1:** Self-interests among stakeholders

	Intrinsic self-interests	Extrinsic self-interests
Community members	– Esteem for their village (special health designations)	– Increased health – Potential for increased income
Community health volunteers	– Self-esteem – Belonging to a group of community advocates	– Leadership development – New skills – Potential for short-term contract work
Paid workers within the community health system	– Increased self-esteem for a job well done	– Improved work performance – Improved efficiency in services
Institutional level of the health system	– Joy at work – Enhanced motivation of employees	– More effective services (enhanced referrals from the community, greater demand for services)

As stakeholders, such as the types listed in Table 1, pursue self-interests and fulfil community goals, the ‘glue’ that binds them toward future goal attainment is gratitude and indebtedness. Equity theorists have shown principles of reciprocal indebtedness are critical to the perception of fairness. Reciprocal indebtedness and gratitude drive inputs in a relationship because individuals modulate their commitment and contributions based on their desire to reciprocate and reduce perceived inequity (Cohen and Greenberg, 1982; Wiertz and DE Ruyter, 2007). Gratitude can expand the arc of the relationship. As gratitude is expressed, an opportunity presents itself to acknowledge indebtedness because the relationship has functioned to fulfil basic needs. These basic needs include growing self-awareness, improved skills and increased knowledge. This motivates stakeholders to contribute to the group from which they fulfil self-interests.

Researchers in relationship marketing draw upon equity theory and observe that gratitude plays an early role in relationship development. Gratitude is transformational in relationships; as gratitude is expressed it functions as a future mediator of trust. The gratitude articulation in recognition of shared power, authority and responsibility can deepen, generating reciprocity cycles as there is a sense of a shared common goal (Raggio *et al.*, 2014). Both indebtedness and gratitude prompt stakeholders to continue working together on community problems and projects, which can continue to fulfil their self-interests, and so on, and continue to generate a commitment to the group’s common goal.

## Putting it together: HCD and a process-based theory of trust

**Figure 2:**
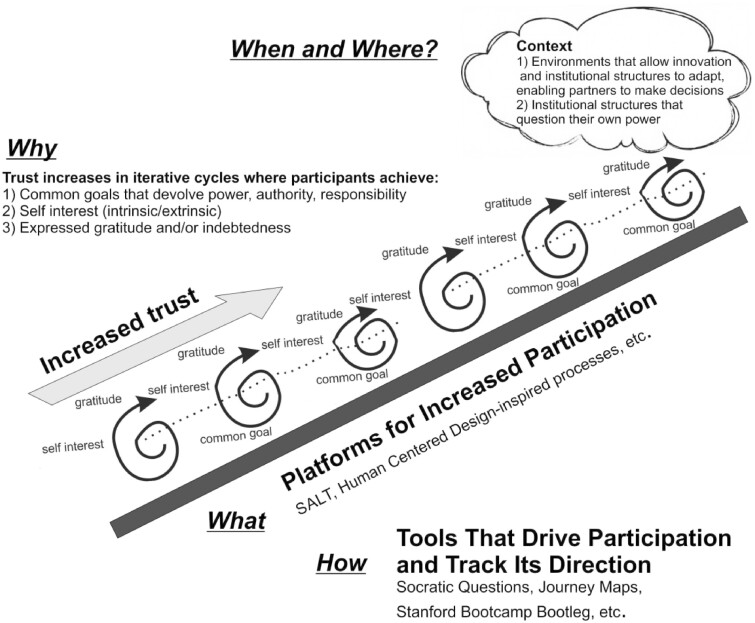
Platforms for increased participation.

Together, a process framework of engagement (HCD), along with a reciprocity-based theory of trust, provides a way of understanding sustained engagement (see Figure 2). It can be used to assess conditions under which an intervention is effective, why, for whom and how, all questions often missing in the HCD literature on population health interventions (Moore *et al.*, 2019).

An HCD platform is the ‘what’ for increasing participation and it uses design tools for ‘how’ stakeholders can meaningfully interact to create community-informed outcomes with strong implementation potential. A training process we developed for close to community health systems stakeholders, SALT (which stands for Stimulate-Appreciate-Learn-Transform and more fully described later in this article) is an HCD platform for the ‘what’ and ‘how’ to occur. The ‘when and where’ refers to context; participatory-driven work can happen within a health system when its institutional structures are open to multi-stakeholder driven innovation. This must involve the institution’s willingness to question their own power and cede it, whenever possible, to stakeholders whom they serve.

The process-based theory of trust is the ‘why’ stakeholders can sustain their work beyond a traditional HCD product or process endpoint. The ‘why’ for understanding sustained engagement is built through the reciprocal process of trust building shown in Figure 1. The three elements of this trust building are displayed in Figure 2 as iterative PDSA cycles. As stakeholders sustain their engagement with each other, we see expressions of gratitude, awareness of fulfilled self-interest and alignment to their common goal to get to the next state change.

As they engage in cycles of reciprocal interaction, stakeholders calibrate and temper their risk in terms of time and energy. This process allows for incremental steps toward trust, allowing stakeholders to build on success or to preserve energy for more productive transactions. It provides a space to pivot on investments, allowing stakeholders to invest while promoting self-interests and preserving one’s sense of fairness regarding inputs in a relationship. That is, cooperative reciprocity cycles over time contribute to increased trust. Yet, if group goals are violated—that is, promises among stakeholders are broken, or the ability to fulfil self-interests derailed—actors move down the HCD platform. Trust also can easily be eroded in environments where structural and/or environmental factors influence group outcomes.

## Methods

In this section, we describe process-based methods and measurement constructs (Mcclintock *et al.*, 1979, Langley, 1999, Langley *et al.*, 2013). We also describe data collection and methods for case study analysis (Mcclintock *et al.*, 1979) as it relates to community health workers’ ability to create positive community change in Kenya relating to social determinants of health. We present data from five Community Health Units (CHUs). These CHUs represent a range of locations from a rural agrarian to peri-urban slum. Each CHU represents a different type of geographic location, income and educational background. The variation of CHU location allowed for a stratified sample of cases for enhanced generalizability, accuracy and simplicity (Mcclintock *et al.*, 1979).

In our region of Kenya, a CHU is made up of about 30 volunteers assigned to a local health centre, serving an approximate population of 5000 (Mccollum *et al.*, 2016). During the SALT workshop, we further subdivided each CHU into smaller working groups or cases for analysis. Our unit of analysis is the case, or group, of 6–8 community health volunteers (CHVs) (*N* = 21 cases). The 21 cases are nested within the 5 CHUs. Longitudinal quantitative data analysis tracked action plan activities from each case (or group of 6–8 CHVs) and the resulting PDSA cycles over 12 months. Thick case description is developed from detailed observations of site visits with each group and recorded conversations of individuals as they participated in focus group discussion at 3, 6, 9 and 12 months.

**Figure 3:**
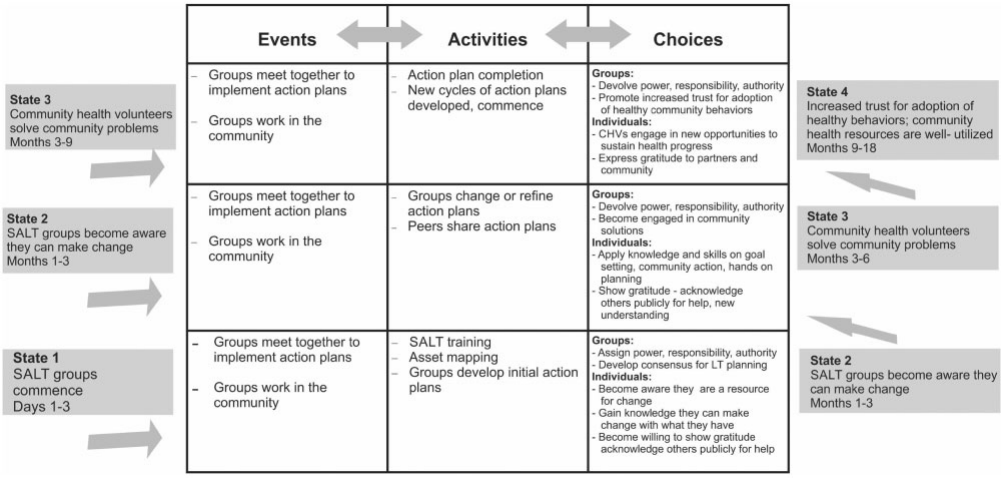
Events, activities and choices in development of state change for process outcomes.

SALT workshop: CHVs operating in a close to community health system participated in a 3-day training, SALT and developed action plans for community change (described more fully later in this paper). CHVs then carried out their work through activities and events, and through making choices about how to build common goals, achieve self-interests and share gratitude/indebtedness (Figure 1). Figure 3 shows how CHVs carry out those events, activities and choices over time, and it includes measurement constructs for assessing state change.

We applied the process of Figure 3 to case clusters within the five CHUs that implemented SALT. Positive changes, occurring among the 21 cases shown in Table 2, included the implementation of self-driven, self-financed projects ranging from hygiene education, table banking, digging soak pits for improved sewage disposal and hygiene, bedbug eradication efforts, sexual and gender-based violence prevention education, school dropout rehabilitation, mentoring for street drug users and hygiene education and surveillance for informal day-care centres and barbers/salons.

**Table 2:** Data collection for cases

Data collection	Community Health Unit Wangu	Community Health Unit Kamere	Community Health Unit Karagita	Community Health Unit Ndeiya	Community Health Unit Kamirithu	Total
Type of community	Rural agrarian	Densely populated informal settlement	Urban flower farm, informal settlement	Small city	Peri-urban area	
Start date (SALT workshop)	March 2016	November 2016	February 2018	July 2018	April 2018	
No. of groups	4	5	4	4	4	21
Number of CHVs	27	25	28	30	30	141
Population served	25 000	18 000	20 000	11 000	21 000	95 000

We selected a cluster of four cases from one community health unit in a peri-urban slum for discussion. One of the four groups in CHU demonstrates ‘outlier’ qualities. The trajectory of this group is one of stopped and stalled attempts at community change and then an eventual change in project direction. This near-failure case, and its relationship to the three other cases in its parent Community Health Unit (CHU), demonstrates how a theoretical framework of understanding (state changes linked to reciprocity cycles of trust building) advances understanding about why a project was able to move forward as the CHVs implemented an HCD process (SALT).

### Data collection

Data collection was taken through multiple means. Qualitative methods included focus group interviews with CHVs and field notes documenting site visits at the 3, 6, 9 and 12 months of marks. Kijabe’s MNCH program staff examined how CHVs developed, modified and implemented action plans over time and sustained engagement as they implemented their projects. Quantitative data included documenting CHV participation, frequency of group meetings and impact in the community or region.

All interviews were conducted in Swahili and in the specific dialect of each of the study locations. Interviews at each study location were recorded, transcribed and translated, with the assistance of translators familiar with the local vernacular.

### Analysis

The conceptual framework shown in Figure 3 was applied to analyse the following themes in the focus group interviews and field notes: CHV choices in building common goals, achieving self-interest and expressing gratitude and indebtedness. This conceptual framework was developed after observing and analysing data from the first two SALT workshops. Upon follow-up at the 18–24 months’ timeframe, all groups continued to drive local change without any external funding or coaching. Individuals in these first two CHUs articulated specific elements of reciprocity cycles and acknowledged the building of trust itself, which is what drove the second phase of this work.

The Kijabe Hospital Ethics Review Committee approved the implementation of the SALT workshop and longitudinal follow-up under a research protocol. All participation was voluntary, consistent with Kenya’s Community Health Strategic goals. This included full knowledge and consent from the local chief and administration.

## Results

### The CHV and CHU program

The partnership in Kenya’s primary care health system-community interface is made up of both health facility employees (local facility nurses and public health officers who function as community health extension workers in addition to other duties) and local community members (unpaid CHVs, who are recognized local leaders). Sub County Health Management Teams supervise front-line staff in multiple facilities with employed Community Health Extension Workers (CHEWs) as well as volunteer CHVs. Annual work plans are developed by the management team and the facility nurses, public health officers. Volunteers are responsible for implementing priorities determined by the hierarchy.

Traditionally, these actors (the community, the government employed staff at the facility and health management teams) have not engaged as equal partners. Rather, where community health units exist, these volunteers were seen as the bottom rung of the hierarchy. While the community strategy in Kenya has been a policy since 2006 (Mccollum *et al.*, 2016), implementation has been spotty and external donor dependent.

### Addressing conditions for change with SALT

The CHVs went through a training process, SALT (Adam and Donelson, 2018a), led by Kijabe MNCH program staff. After the 3-day workshop, MNCH program staff conducted a series of action-oriented follow-up coaching sessions throughout a 12-month period that enabled local stakeholders to define health action plans and implement, measure and be accountable to them in their communities, using only locally available resources. In the training, participants learned these locally available resources are those they already have or can create with their own hands. For example, compost is a locally available resource one CHU group created through a waste clean-up they performed in the community, which they later turned into fertilizer. This became an income-generating opportunity when they found local fisherman digging for worms in the compost pile, which they said they could sell for 100 shillings per cup. Another example of locally available resources was household burlap bags that would have been thrown out. One CHU group turned them into hanging vegetable gardens in low-income housing. A third example was hot water, used to eradicate bedbugs, rather than expensive chemicals.

The SALT model utilized Kenya’s community health strategy but differed in that the goal was to build partnerships that altered the traditional hierarchical structure, providing the community with a stronger voice for their choices.

Selection of the volunteers (CHVs) followed Kenya’s strategy for community health services. The Kenyan protocol required a nomination process that is driven by their community and the local leadership. Selection criteria include permanent local resident status, the respect of the community and may include the ability to read and write. These volunteers know they have a responsibility which is articulated during the recruitment process. A council of local elders, along with the local facility nurse and the region’s community health extension worker (paid staff), came together and participated in *barasa*, which in Swahili means ‘long sitting’. This group consensus process, which involves discussion, produced nominations of those who were believed to be the best representatives for the community.

Once identified, those representatives then passed a SALT facilitator check to ensure the volunteers adequately represented a broad range of community sectors (i.e. disability, youth, health, business) and geography. In the past, subcounty health management teams recognized some community health workers had not been well-represented by geographic regions and sectors, which could put projects at risk because of the lack of broad participation. This is a risk well-recognized in the literature on voluntary organizations. ‘Oligarchic rule’ has been shown when the minority, feeling they are the only ones committed to a cause, satisfy their own needs and interests and are reluctant to change if they perceive changes threaten their own interests (Harriss, 2002).

Finally, MNCH program staff vetted the CHVs to ensure they had the time for the project. It required approximately three full days of training and about 4–8 h of time per week thereafter. CHVs continued this amount of work indefinitely, as long as they wanted to remain volunteers, as their commitment. CHVs split into self-governing groups (within the CHU) to identify community action plans. They then met every 3 months together with MNCH program staff to see what the groups had done. MNCH staff encouraged the groups to develop, implement and revise the plans as needed.

### Case study: community conditions prior to intervention in the peri-urban slum CHU

The CHU one of several in a peri-urban slum with a population of approximately 220 000. Families working in the area are mostly employed by flower farms, although some work for small scale fisheries and small businesses. A quarter of the residents are temporary dwellers brought either by family members or friends to work there. Roads are dusty, drainage is poor and residents lack access to tap water; taps are locked for rationed use.

CHVs in the four groups chose to address several social determinants of health within four settlements of the larger area. Groups 1, 3 and 4 were interested in addressing frequent breakouts of diarrhoea and cholera. In the past, an external NGO had funded a program to introduce tippy taps or rudimentary handwash stations, in many households of that community so as to improve opportunities for handwashing and reduction of disease. Yet, the tippy taps had fallen into disrepair. None of them was working. Although an apparently simple solution—one relying on locally available materials, but external donors and funding—the community had not owned the solution. CHVs, through the SALT training, decided the project was worth revisiting, with each of the three groups addressing different strategies. They decided a hand hygiene education, outreach efforts and a tippy tap repair program met SALT principles: it required only locally available materials and was easy to implement and available to the entire community. Group 2 was concerned with chronic poverty and chose to address this through targeting school drop outs and initiating a table banking project (a savings and credit cooperative) to help the group members start businesses.

### Process of change

#### Three-month visit

The MNCH staff followed up with CHVs in each of the four groups to discuss progress made in implementing the process model shown in Figure 3. Table 3 shows how each group took action and whether their choices fulfilled or disrupted trust-building conditions of reciprocity by the 3-month mark.

**Table 3:** Three-month CHU visit: project progress and conditions for reciprocal trust

	Events/activities	Choices for reciprocal trust building		
Group no.	Develop and implement action plans	Common goal - Joint power, responsibility, authority - Consensus on decision-making	Self-interest Awareness they are capable of receiving intrinsic and/or extrinsic benefits	Indebtedness/gratitude - Willingness to show gratitude/indebted to others for help
1	20 households with new tippy taps (one with a new bathroom)	Common goals fulfilled. CHVs made unanimous decision on their work plan. They visited 20 household by working in pairs, with 4 individuals each visiting 5 households.	Self-interest identified. CHVs helped households make change with their own resources (four property owners used their own resources to build tippy taps).	Gratitude expressed. Community members, including the police who lacked hand wash facilities, commended CHVs for their work, encouraging them to continue working together.
2	Work with 15 school dropouts to return to school; create table banking for a group of 11	Common goals fulfilled. CHVs worked out table banking strategy, bylaws and interest and asked CHU to serve as accountability partner. CHVs followed up with 10 dropouts, 9 of which returned to school.	Self-interest identified. CHVs developed their new way of earning resources through table banking.	Indebtedness expressed. One CHV noted: ‘You know we could not add more members (to our table banking) until we learn from each other, know each other and more importantly, trust each other’.
3	Six kindergartens, six landlords trained in hand washing Gender and sexual based violence—three cases	Common goals articulated. Although three CHVs dropped out, three worked together, changing strategy from more staff-intensive soap making plan to less staff-intensive door to door outreach.	Self-interest identified. One CHV noted: ‘I was the kind of person who would not do a thing if there is no income… After the training I realized that there is more than just monetary value…Now compassion is my greatest drive in community work’.	Gratitude expressed. One CHV noted: ‘I cannot equate the work we do to…monetary value. It is far worth more than that. If I would ask people to pay me for what I teach them when I visit their homes…most of them cannot afford it. A thank you is enough for me’.
4	Construction of one tippy tap	Common goals not articulated. Group does not share power (the leader made all decisions); group is not meeting regularly.	Self-interest not identified as part of the project. The leader was motivated by external compensation unavailable to the group.	Gratitude and indebtedness not expressed at CHU visit. Leader expressed they were entitled to more resources.

As shown in Table 3, Groups 1, 2 and 3 made significant progress in their action plans. They shared how their choices facilitated the movement of the projects. However, Group 4 struggled. When the group’s chairman took the CHU staff on their site visit to visit a tippy tap, MNCH staff discovered the chairman had built it the day before the meeting. In discussion, it was clear the group had not been meeting over the 3 months. The group members admitted none had wanted the leadership role and had argued over who would end up being in charge. The chairman stated he would take the steps necessary to engage the community in a handwashing campaign. The MNCH staff listened, affirmed that they as a group possessed the resources they needed to make the change happened, and left, noting they would return in 12 weeks.

#### Six-month visit

Three months later, the CHU staff returned for site visits with each of the groups. Table 4 shows how each group took action, and whether their choices fulfilled conditions of reciprocity between the 3- and 6-month mark.

**Table 4:** Six-month CHU visit: project progress and conditions for reciprocal trust

	Events/activities	Choices for reciprocal trust building		
Group no.	Develop and implement action plans	Common goal - Joint power, responsibility, authority - Engagement in community solutions	Self-interest - Apply knowledge and skills in the community to receive intrinsic and/or extrinsic benefits	Indebtedness/gratitude - Show gratitude/indebtedness to others
1	Continued hand washing education and tippy tap construction	Common goals fulfilled. CHVs continued to work using the same plan.	Self-interest met. CHVs continued to apply knowledge and skills in the community.	Gratitude expressed. Community continued to commend CHVs for their work, which encouraged them to continue working together.
2	Community education on school dropouts; table banking	Common goals fulfilled. Table banking operated for three months with 10% interest. CHV observed, ‘What we have done, (we) could not do alone, but we as a group have taken long notable strides’.	Self-interest met. CHVs shared how the project met their needs for friendship and improved confidence.	Gratitude expressed. In their meeting with the CHU, CHVs expressed appreciation for each other and the group that was undertaking the hand washing effort.
3	Hand washing Gender and sexual based violence	Common goals fulfilled. CHVs continued to work using the same plan.	Self-interest met. CHVs continued to apply knowledge and skills in the community.	Gratitude expressed. Community continued to commend CHVs for their work, which encouraged them to continue working together.
4	Construction of three school tippy taps	Common goals not well articulated. Group does not meet regularly. They repaired three tippy taps, but were unable to initiate a handwashing campaign.	Self-interest partially met. One noted, ‘Hand washing has helped reduce diseases in my own family…just as (in) the community’. The leader continued to be motivated by external compensation.	Gratitude partially expressed at CHU visit. One CHV congratulated Group 2 on their progress. At end of meeting leader continued to state they were entitled to more external resources for transportation.

Groups 1, 2 and 3 continued to make steady progress in implementation, as shown in Table 4. They continued to address elements of the reciprocity cycle to become more effective in their work. In their follow-up discussions with CHU staff, Group 4 focused on the implementation challenges they faced in repairing three schools’ tippy taps: the soap was frequently stolen, children would break the tippy taps so they could see the water trickle, and the USAID posters they had put up reading ‘Wash your hands here’ had been torn down. They asked CHU staff if it would just be easier to mix liquid soap in the water—to which the CHU staff replied that would just leave people who use them with soapy hands.

Seeing the difficulty Group 4 was having, MNCH staff convened a meeting in which Group 2 was able to share their work about their table banking project and student dropout prevention program. CHUs from Group 2 discussed details about the families of school drop outs they visited, how they worked with the families to get their birth certificates and waive school fees to get them back to school, and the intricacies of how they established the table banking strategy and recruited new members. They shared how they had moved from focusing on the implementation challenges to solutions: ‘I used to ask myself why there are so many school drop outs, and I did not know how to approach the problem. In the group, I have gained the confidence to start bold conversations with the parents of these drop outs in a bid to get them back to school’. Another shared, ‘Being in this group opened my eyes to seeing that is not just the overlying problem that we see: at times it is from a deeper cause and I have to dig to get at it’. This made an impression on Group 4, one of whose CHVs noted, ‘I wish to congratulate Group 2 for their progress. Clearly, they are many steps ahead. Keep it up’.

At the end of the meeting, the chairman of Group 4 stated the group was entitled to more external resources. He told the MNCH staff they needed to provide more money (transport reimbursements) if the CHVs were to continue with the work. The MNCH staff held fast and reaffirmed the SALT principles from the training: the CHVs had all they needed to make change. The MNCH staff made plans to return in 3 months.

#### Nine-month visit

Groups 1, 2 and 3 continued to make progress in implementation and in building trust with each other and the community. Upon their return to Group 4, MNCH staff were surprised to a change: six of the CHVs were present, and only two were absent and had sent their apologies. They reported on their progress visiting six schools, where they repaired six more tippy taps and delivered hand washing education through the head teachers. In the discussion, the chairman was quiet, while other members discussed their work and school visits. They had educated schoolchildren who were now encouraging their parents to wash their hands. The CHVs described their effort to begin making and selling soap to the schools. The schools were asking for their soap frequently enough to encourage the CHVs to check regularly to make sure the tippy taps were working well. Group 4 had also expanded their efforts beyond schools to work with landlords who were now providing water in their plots for hand washing. The CHVs observed that teachers were reporting incidences of diarrhoea were on the decline.

#### Twelve-month visit

The MNCH staff followed up for a final report with each group. The impact of their work is summarized in Table 5.

**Table 5:** CHV impact at 12 months

Group no.	Impact at 12 months
1	76 tippy taps constructed and remain functional with random site visits 80 households visited (20 for each of the 4 CHVs) with handwashing education Two schools educated in handwashing Three churches educated in handwashing One new tippy tap at bus stop
2	21 children back to school Table banking enables CHV takes two loans to expand grocery store and stock Table banking enables CHV to purchase cow feed Table banking enables CHV to harvest and sell hay to pay for school fees
3	30 tippy taps built in households, churches, schools and hospital CHVs pooled own funds to start making, selling soap CHVs carry out handwashing campaign and sell toilet paper, diapers and household items at discount and on credit so they are affordable to the community, unlike local supermarkets Three cases of sexual and gender based violence identified and one case prosecuted
4	11 schools educated on handwashing 9 tippy taps constructed CHVs make, selling soap to schools

Dynamics among Group 4, which had started out with little trust among its members, had changed. The group described how they had now reached all students in the area schools—5 additional schools since the last visit. They reported seeing the impact of decreased cases of diarrhoea. Schools now requested that they teach not only children but also teachers on hand hygiene practices.

Using SALT principles (HCD platform) and continuing PDSA cycles in their work, they were finally able to address the many implementation problems they had brought up at the 6-month mark. Now, schoolchildren and teachers were doing things correctly. Before, most had only washed only their fingers; some schools had only washed the hands of many children at the same time in a single basin or not washed at all before lunch despite singing the kunawa hand washing song. Others had mixed the soap and water in the tippy tap, making rinsing ineffective.

All of the groups discussed plans or steps they already were taking to carry out their next phase of work. These groups continued to sustain their impact without funding. As a CHV noted at the 12-month debriefing meeting, ‘I previously thought that the SALT program was all about giving us money in order to keep the community clean. Little did I know that I was going to help the community without spending money?’

At the year mark, Group 1’s CHVs had decided to grow gardens on their porches, using gunny sacks and grey water, since water continued to be in short supply. They planned community outreach and education on how to create the gardens to improve nutrition and save money, as they were already seeing in their households. Group 2 planned to continue its table banking. They also had the respect of the community as a group that was helping students return to school. Group 3 discussed how their business made and sold soap, as well as how wholesale toilet paper and diapers brought them new income that paid school fees and saved money. Group 4 shared they would continue to sell soap. One CHV noted: ‘(We) cannot (stop) the project because we have tasted the benefit’.

## Results

**Figure 4:**
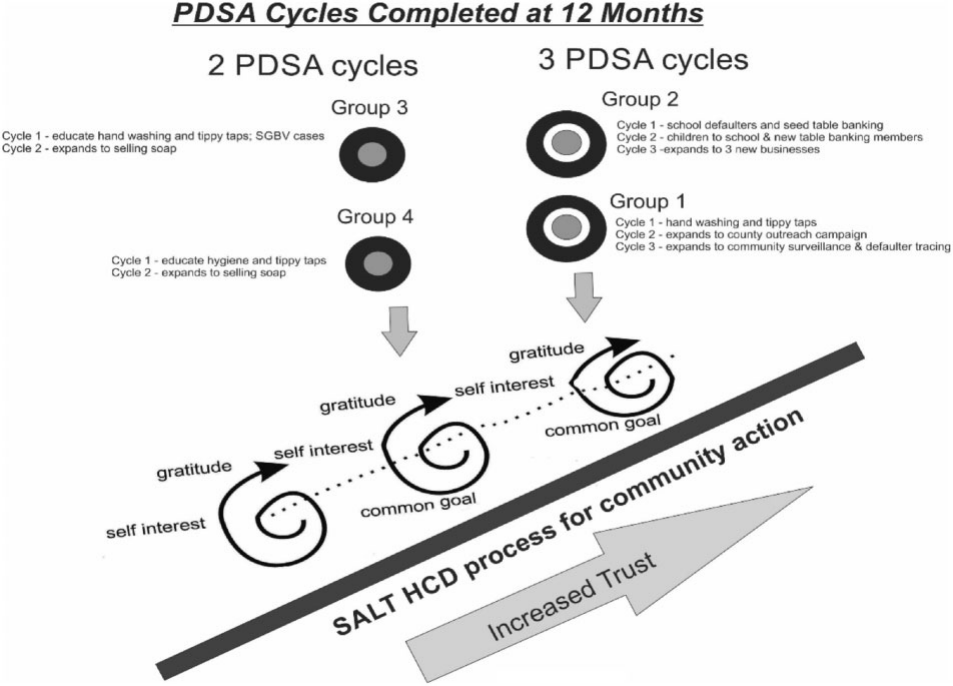
PDSA cycles completed at 12 months.

All groups in the case cluster demonstrated progress and exceeded goals. By the 12-month period, they completed between 2 and 3 Plan-Do-Study-Act cycles and in the process completed reciprocity cycles building trust, shown in Figure 4.

A theory of trust building, operating on the HCD SALT platform, provides an iterative process to assess sustainability, mediate implementation problems and frame measurement constructs. As for sustainability, all four groups continued their work past the 12-month period, using their own resources. Even Group 4, where members seemed to lose trust in the process over the first 6 months, began to work collaboratively after near failure.

We do not know the factors that enabled Group 4 to come back together. It is possible that re-engagement was culturally stimulated by shame at what Group 2 was able to accomplish (a form of cross-learning). It may have been motivated by leadership frameworks that matured within the group, such as by actors who may have developed clearer boundaries over time. Ostrom and Walker (2003), for example, have written much about how effective boundary-setting conditions help actors within a group more effectively co-produce and manage common resources in a sustainable way. While we do not know what conditions caused them to return together to jump start a near-failed process, we do know that upon re-engagement CHU staff documented evidence of the iterative reciprocity cycle (common goal, self-interest and gratitude). Efforts continued without any additional funding through the 18 months follow-up.

All four groups were able to mediate implementation problems common in their work by drawing upon HCD-informed practices of deep observation and using their own resources. Group 4, at the 6-month mark in their project, identified many implementation problems. By month 12, however, they persisted in helping schoolchildren and schools adopt proper handwashing practices and were able to maintain functional tippy taps during their soap sales visits.

The HCD process also enabled the MNCH team to experiment with and test measurement constructs iterating through the first two SALT workshops and testing the measurement constructs in SALT workshops 3, 4 and 5 as shown in Tables 3, 4 and 6. These measurement constructs—especially the presence of gratitude—draws from the empathy elements in HCD approaches. They help articulate the ways activities, events and choices drive the state change processes described in Figure 3. These measurement constructs show how CHVs were able to initiate and continue work that fulfilled self-interest (earned income and community status), achieve a common goal with consensual, shared authority, responsibility and power, and continue the feedback loop by expressing gratitude and indebtedness.

**Table 6:** Nine-month CHU visit: project progress and conditions for reciprocal trust

	Events/activities	Choices for reciprocal trust building		
Group no.	- Action plans completed/new cycles developed	Common goal - Joint power, responsibility, authority - Engagement in community solutions	Self-interest - Engage in continued opportunities to sustain health progress and receive intrinsic and/or extrinsic benefits	Indebtedness/gratitude - Express gratitude/indebtedness to others
1	55 new tippy taps and 15 new latrines; worked with county government sanitation program	Common goals fulfilled. Group members know each other’s work and have worked jointly to conduct outreach.	Self-interest met. They have continued with their plan in teaching people how to use handwashing and tippy taps.	The CHU reported the CHVs are “happy they are involved in the community” and “calm and collected” as they do their work.
2	9 school dropouts back to school; table banking group grows	Common goals fulfilled after changing leadership; former leader had not shown to recent meetings. They reported being proud of table banking and school dropout interventions.	Self-interest met. They are benefiting from table banking loans, which are about to mature for some members.	Gratitude expressed. The chief and neighbours know them, and they say they are thankful to be addressing deeper community problems than when they started.
3	School visits on Gender and sexual based violence	Common goals fulfilled, but the group reconfigured around their interest areas, with half implementing one action plan and half implementing a second action plan.	Self-interest met. They are getting county-level training on sexual and gender based violence. One CHV started a business and used SALT training as an incentive to start; another started a soap-making business.	Gratitude expressed. The group is grateful cases of diarrhoea are on the decline. One CHV noted: ‘Nowadays we do not have frequent cases of illness and outbreaks like we had before we started educating people on proper hygiene. Members of this community have actually labelled us “village doctors”’.
4	Construction of tippy taps at six schools, soap making, and handwashing campaign	Common goals fulfilled. Chairman was silent at CHU visit, as other members described how they visited six schools in pairs to educate them on handwashing and sell soap.	Self-interest met; they had direct monetary gain from soap making. Once CHV noted, ‘We never disturb our CHEW…to get money. We can sustain ourselves’.	Gratitude expressed. They reported being indebted to Group 2 for giving them an ‘awakening call’ to do the work.

Using an HCD process (inspiration, ideation and implementation) separate from the SALT workshop, the MNCH team refined measurement constructs, articulating the manifestation of self-interest, and gratitude and indebtedness using their deep knowledge of the vernacular language and the cultural context.

## Discussion

HCD is a powerful framework for promoting stakeholder engagement in close to the community health systems. However, it cannot on its own generate sustained change. Despite enthusiasm for HCD in social innovation, there is limited empirical evidence HCD can impact health outcomes beyond short-term interventions (Bazzano *et al.*, 2017). Short-term interventions, while effective for product development or process improvement, fall short of addressing sustained change in health systems where sustained collaboration and shared leadership are important (Eder, 2013; Moore *et al.*, 2019). Better use of theory is required for use of HCD in health systems, if possible with an explanation of causal mechanisms, to understand population-level health outcomes (Bazzano *et al.*, 2017).

In fieldwork, the authors developed an HCD platform for stakeholder engagement. Using HCD processes, the research team mediated implementation problems and framed measurement constructs for assessing longitudinal behaviour patterns. Critical reflection on the ‘why stakeholder engagement was sustained’ allowed patterns of behaviour to emerge from the data. Reproducible patterns of behaviour were organized and compared to existing theories of behaviour change, ultimately culminating in a theory of reciprocity cycles for trust building. HCD not only provided a platform for stakeholder engagement, resulting in co-production of knowledge, but also supported ongoing critical reflection to address the ‘why’ the process could be sustained. Linking HCD to theory, demonstrated in fieldwork, adds explanatory power as well as pragmatic value to health system change.

This study has limitations. As embedded researchers, internal evaluators are subject to bias. They can inadvertently lead participants to over-report ‘good’ behaviours or results and underreport ‘bad’ ones. For example, regular MNCH staff visits at 3-month intervals may have acted as a booster to keeping groups on track with their projects. In future work, this potential effect could be mitigated by making fewer visits (at 6 month intervals) and asking cohorts to track their own work in the interim, so as to determine the impact of observation versus MNCH intervention. However, the fact that the groups sustained their work past the 12-month period of observation, and often beyond 18 months of any MNCH observation, points to evidence of sustained stakeholder engagement.

The heterogeneity of case studies also can mitigate the potential for bias. By using multiple cases and multiple data sources or methods of observation, we follow the multi trait-multi method logic of Campbell and Fiske (1959) in order to reduce threats to internal validity. Participant observation, in-depth interviews and repeat contacts with the groups gave the authors intimate knowledge of the social forces they were studying. The CHVs themselves, through their activities and their own interpretations of what was taking place, also served as a check to bias.

The novel approach of linking HCD to theory means there are few if any other examples of this approach. Time will tell if this approach gains favour. HCD itself may be further limited by practitioners who take a ‘consultancy’ approach to problems, thereby limiting essential stakeholders like CHVs to token involvement, falling far short of true engagement that might sustain implementation as well as promote their perspective in addressing barriers to implementation of an intervention (Arnstein, 1969; Tritter and Mccallum, 2006; Rifkin, 2014).

We also recognize that our example of trust building as a theory linked to HCD is limited by context. Institutions with top-down decision-making approaches, that is, ones unsupportive of innovation at the grass roots, will not embrace HCD. Trust building requires elements of a supportive context. This requires the institutional structures of health systems to question their own power and cede it, whenever possible, to those they serve. Support for innovation can be limited in institutions that value highly structured hierarchical decision-making, such as Kenya’s Ministry of Health. These organizations do not easily adjust to distributed leadership models and are at risk of default into tokenism over true engagement. Collaborative processes are required for trust to develop. That demands a supportive context, even if that supportive context functions within a microcosm of the system. Gains in trust can be only be made when patient voice and agency are fully supported by the institutions that serve them.

## Conclusion

We demonstrated how HCD, when linked to theory, can be harnessed for understanding process change. We established how a process model can address short-term to longer-term measurement constructs, provide a longer-term framework to address implementation problems and drive sustainability.
